# Aspects of Embryonic and Larval Development in Bighead Carp *Hypophthalmichthys* *nobilis* and Silver Carp *Hypophthalmichthys molitrix*

**DOI:** 10.1371/journal.pone.0073829

**Published:** 2013-08-14

**Authors:** Amy E. George, Duane C. Chapman

**Affiliations:** Columbia Environmental Research Center, U.S. Geological Survey, Columbia, Missouri, United States of America; Institute of Marine Research, Norway

## Abstract

As bighead carp *Hypophthalmichthys* *nobilis* and silver carp *H* *. molitrix* (the bigheaded carps) are poised to enter the Laurentian Great Lakes and potentially damage the region’s economically important fishery, information on developmental rates and behaviors of carps is critical to assessing their ability to establish sustainable populations within the Great Lakes basin. In laboratory experiments, the embryonic and larval developmental rates, size, and behaviors of bigheaded carp were tracked at two temperature treatments, one “cold” and one “warm”. Developmental rates were computed using previously described stages of development and the cumulative thermal unit method. Both species have similar thermal requirements, with a minimum developmental temperature for embryonic stages of 12.1° C for silver carp and 12.9° C for bighead carp, and 13.3° C for silver carp larval stages and 13.4° C for bighead carp larval stages. Egg size differed among species and temperature treatments, as egg size was larger in bighead carp, and “warm" temperature treatments. The larvae started robust upwards vertical swimming immediately after hatching, interspersed with intervals of sinking. Vertical swimming tubes were used to measure water column distribution, and ascent and descent rates of vertically swimming fish. Water column distribution and ascent and descent rates changed with ontogeny. Water column distribution also showed some diel periodicity. Developmental rates, size, and behaviors contribute to the drift distance needed to fulfill the early life history requirements of bigheaded carps and can be used in conjunction with transport information to assess invasibility of a river.

## Introduction

Bighead carp *Hypophthalmichthys* *nobilis* (Richardson) and silver carp *H* *. molitrix* (Valenciennes, the bigheaded carps) are invasive species in North American waterways, and are currently poised to enter the Laurentian Great Lakes, where they could damage a fishery valued at $7 billion annually in the United States portion alone [1,2]. Assessments of the potential of bigheaded carp establishment have been published that are partially reliant on the early developmental rate of the carps [3,4]. However, there is still uncertainty about developmental needs and rates for embryonic and larval bigheaded carps, especially with regard to water temperature. Early larval behavior and swimming capacity, information which is vital for modeling larval dispersal [5], are also sparsely documented [6,7].

In their native systems, bigheaded carps normally live and feed in floodplain lakes and low water velocity areas, but they spawn in turbulent portions of large rivers [3]. Adult bigheaded carps introduced into large lentic systems such as the Caspian Sea [8] or large reservoirs [9] live and feed in lentic waters, including open water areas, but move into large rivers to spawn. To date, three robust bighead carp have been captured from Lake Erie, all from the lake itself, not from river tributaries [10]. Thus, while ecological effects of establishment are most likely to be experienced in the lake environment, the adequacy of available spawning rivers is highly important in determining the invasibility of the Great Lakes Basin by bigheaded carps.

The eggs and early larvae of bigheaded carps drift in the current; they are more dense than water and are thought to perish if current and turbulence are insufficient to keep them from settling to the bottom before they reach a stage at which they can swim [3]. It is thus thought that a minimum length of river is required for development of eggs and larvae. Required river length has been most commonly cited as anywhere from 80 to 100km [3,11,12], but these numbers are based on observations of locations where the fish are established, not by field or laboratory tests or models. It is logical that required length is largely dependent upon flow regime, hydraulic conditions, and temperature of the river, as well as developmental rate and behavior of the carp.

Early developmental stages and some notes on behavior of bigheaded carps have previously been described [7,13], along with preliminary information on the developmental rate of each species [13]. Developmental rate in fishes and other poikilotherms is highly correlated with temperature, and small changes in temperature can drastically affect developmental rates [13,14], which affects the minimum required drift period and thus minimum river length. Knowledge of developmental rate and early life behavior is critical to efforts to model rivers to determine their suitability as spawning and recruitment sites of bigheaded carps [4]. Such information is also useful for determination of bigheaded carp spawning locations [15]. Given knowledge of spawning locations, models like that of Deters et al. [15] could also be used with development and behavior information to determine the region at which larvae would leave the drift and move into nursery habitats.

The first objective in this study was to estimate the developmental rates of bighead and silver carps based on a combination of time and temperature. Secondly, we sought to examine the utility of egg size as a characteristic for identification and aging. The third objective was to describe swimming behavior in the pro-larval period.

## Materials and Methods

### Ethics statement

The study plan was approved by the Columbia Environmental Research Center Institutional Animal Care and Use Committee and conforms to relevant national and international guidelines. All field collection of adults was carried out using trammel nets under a Missouri Department of Conservation permit.

### Aquarium setup

All water used was Columbia Environmental Research Center (CERC) well water, which is non-chlorinated water. For all experiments, two temperature treatments were used to culture eggs and larvae in the lab. Each temperature treatment consisted of 10 aquaria that were each equipped with a modified MacDonald hatching jar (45 cm tall and 13 cm internal diameter) and a small submersible pump to recirculate water from the aquarium into the hatching jar, and to provide an upwelling current to keep the eggs suspended in the water column within the jar. Jars were equipped with surface screens to prevent any eggs escaping into the aquarium before hatching. All aquaria were provided with a slow flow-through of CERC well water. Temperature was monitored continuously with HOBO temperature loggers (Onset Computer Company, model Pro-v2) recording at 15 minute intervals.

The temperature range of 19-23°C was selected to reflect conditions that are common in mid-continent rivers, and are not intended to represent the thermal minima, maxima, or optima. In 2010, the “warm” treatment, temperature was maintained above ambient using a heated water bath. In the “cold” treatment, temperature was maintained by room temperature. The bighead carp “cold” treatment was maintained at 20.1° C (SD = 0.39°C) and the “warm” was 22.3° C (SD = 0.15°C). The silver carp “cold” treatment was 19.6° C (SD = 0.17°C) and the “warm” treatment was 22.5° C (SD = 0.10°C). In 2011, all temperatures were maintained by use of heated or chilled water baths. Using water baths, a constant temperature of 18.9° C (SD = 0.18°C) was maintained for “cold” temperature in all experiments in 2011 and “warm” water temperatures were maintained at 23.0° C (SD = 0.49°C) for bighead, 22.5° C (SD = 0.21°C) for the first silver carp experiment, and 22.2° C (SD = 0.09°C) for the second silver carp experiment.

### Spawning details

During assessment of spawning readiness and while waiting for ovulation, brood stock were held at CERC in 1,500 L flow-through fiberglass tanks supplied with CERC pond water at 21–26°C. Fish were evaluated for spawning readiness upon arrival or capture. Fish were anesthetized with tricaine methanesulfonate (MS-222), after which males were evaluated for presence of milt after gently applying pressure to the abdomen and females were evaluated by inserting a catheter into the urogenital pore and collecting egg samples. Eggs were placed into a clearing solution (60% ethanol, 30% formalin, 10% acetic acid) and the location of the germinal vesicle was used to determine if the female would be used for hormone induction.

Doses and timing for hormone induction were based on size and species using data from Jhingran and Pullin [16]. Females received an initial muscular injection of human chorionic gonadotropin (HCG, 200 IU/kg for bighead carp and 600 IU/kg for silver carp), and a resolving dose of 4 mg/kg carp pituitary gland was given six hours later as an intraperitoneal injection. Males received a 4 mg/kg intraperitoneal carp pituitary injection 16 hours before the expected ovulation period. Milt was collected into plastic tubes and stored in beakers on ice prior to fertilization. Milt quality was evaluated by checking the percent of active sperm and duration of motility.

Thirty minutes after initial release of eggs at the time of expected ovulation, the eggs were stripped into a bowl by applying pressure to the abdomen. Oocytes were fertilized with pooled milt from males by the dry method [17] for one minute, then rinsed and placed into a water bath for a 30 minute water-hardening period. Two water baths were used, matching the temperature in the hatching jars where the eggs were stocked.

#### 2010

On June 11, 2010, adult bighead carp were obtained from Osage Catfisheries, Osage Beach, Missouri, USA. Two female bighead carps, 7 kg and 8 kg, and three males (6.5, 7.0, and 8.75 kg) were selected for hormone induction, and received initial injections on June 13, 2010.

On June 28, 2010, adult silver carp were captured in the Missouri River. One female silver carp, 3.75 kg, and two males (3.75 and 4.0 kg) were selected for hormone induction and received initial injections on June 28, 2010.

#### 2011

For the first silver carp spawning in 2011, adult silver carp were obtained from the Missouri River in 2010 and held in research ponds over-winter. On May 23, 2011, two males (4.25 and 6.25 kg) and two females (4.75 and 6.0 kg) were selected for hormone induction. Ovulation occurred only in the 4.75 kg female.

Adult bighead carp were obtained from Ted Shanks Conservation Area, Missouri, on June 7, 2011. Three females, 11.5, 13.75, and 14.75 kg, and four males (7.8, 8.0, 8.5, and 9.0 kg) were selected for use. The largest female took slightly longer to spawn than the other two, so while the eggs were cultured, the resulting larvae were only included in behavioral studies.

For the second silver carp spawning of 2011, adult silver carp were obtained from the Ted Shanks Conservation Area on June 15, 2011. Two females (8.0 and 11.5 kg) and three males (6.5, 8.0, and 10.0 kg) were selected for use.

After 30 minutes, 50 mL of eggs were placed into each of the hatching jars (approximately 3500 eggs), with 10 jars per temperature treatment. Water flow was adjusted so that eggs were kept in suspension in the water column within the jar, but did not impinge on the screen lids. Because eggs changed in buoyancy throughout their first two hours of development, it was necessary to frequently adjust flow rate during that time to avoid impingement of eggs on the screen surface. During hatch, screens were removed and fish were allowed to escape hatching jars into the aquarium. Pumps and hatching jars were then removed, and larvae were maintained in aquaria through at least six days post-fertilization. Larvae had not transitioned to exogenous feeding during the experiment, so no food was offered. Water-quality indicators (total dissolved solids, ammonia, pH, dissolved oxygen, alkalinity, and specific conductance) were measured in jars and tanks before stocking, after hatching, and again following breakdown of the experiment.

#### Developmental stage quantification

Because later stages were longer in duration, intervals at which evaluations were made increased with time. For the first four hours following fertilization, three to six eggs from each temperature treatment were removed from hatching jars by a suction device at 15 minute intervals. After four hours, eggs were removed at 30minute intervals until hatch. After hatching, three or more larvae from each treatment were removed from tanks at 4–8 hour intervals, using either a dip net or a suction device. Samples were removed from alternating jars and tanks in sequential order. Eggs and larvae were examined and photographed under a Nikon SMZ 1500 stereoscopic microscope (7.5x–112.5x total magnification) with camera attachment (Nikon DS-Fi1, 5 megapixel), noting time since fertilization, developmental stage and any abnormalities. Measurements of live egg membranes and larval fish total length were taken using Nikon Elements software. Because eggs are not usually perfect spheres, reported egg measurements are the mean of two perpendicular diameters.

MS-222 was used to anesthetize larvae for live photography. After live photography, eggs and larvae were preserved in 10% buffered formalin in 25 mL scintillation vials. Embryos and larvae were assessed for developmental stage and photographed again after preservation, and egg membranes were removed for image clarity as necessary.

Timing for developmental stages was based on recorded time of the first individual of each temperature treatment found at a particular stage. All stages were based on definitions of Yi et al. [7], and were not counted until the stage could be considered complete (for example, a larva would not be designated as stage 36 (the melanoid eye stage) until the eye was completely darkened). Photographic documentation of developmental stages is provided in the appendices of Chapman and George [13]. No attempts were made to quantify percentage of mortality or abnormalities at any stage.

#### Cumulative Thermal Units calculations

Cumulative Thermal Units (CTU), also known as degree-days or temperature units, is a method designed to calculate the relation between temperature and developmental time in poikilotherms. While a zero degree temperature is occasionally used for a base value, use of a thermal minimum is found to increase accuracy of calculations [14,18]. Thermal minimum is intended to be temperature at which development ceases [19,20], and CTU should be predictive of developmental stage. Thus, the following equation was used for quantifying developmental rate:

CTU = t(T_c_−T_min_), where t = time in hours, T_c_ = treatment temperature in degrees Celsius, and T_min_ = thermal minimum in degrees Celsius.

Using all experimental data for each species, CTU (summation of hourly mean temperature) was computed iteratively for each developmental stage, by using a value for T_min_ from 0.0°C to 15.0°C in 0.1°C increments, in order to determine the value of T_min_ where the CTU value had the lowest variance for all developmental stages. Variance was tested using a one-way ANOVA with stage as the parameter. The main purpose was to obtain the pooled variance and determine the coefficient of determination (R^2^). To obtain the best fit, T_min_ values are reported as separate values for embryonic stages and larval stages.

#### Behavioral observations

Soon after mass hatching (all trials in 2011 – one bighead carp and two silver carp), larvae were taken from the experimental tanks that most closely matched the temperature in the swimming tubes to avoid acclimation effects or temperature shock. Ten to twenty larvae were placed in each of two swimming tubes and allowed several hours for fish to acclimate. In the 2 m x 10.2 cm internal diameter clear PVC swimming tubes, water column position, swimming patterns, and ascent and descent rates were observed at 12 hour intervals (at approximately 12am and 12pm) to capture differences in diel cycle. For each interval, mean ascent and descent rates were calculated. All experiments were performed with overhead lights on a timer (14 hours light: 10 hours dark) to mimic diel patterns in an otherwise light-tight room. Flashlights provided any directional lighting necessary to locate fish. Efforts were made to avoid shining flashlights directly on the larvae, because advanced larvae sometimes reacted to direct light by sinking. Temperature was monitored at top and bottom of tubes using HOBO temperature loggers (Onset Computer Company, model Pro-v2) recording at 15 minute intervals. Temperature in the swimming tubes was controlled by ambient room temperature. Determinations of larval stage were made by visual inspection. Observations of larvae in these containers continued until after gas bladder inflation, a period of at least five days.

#### Statistical comparison

An ANCOVA was performed on diameter of eggs past water hardening. The covariate was adjusted temperature to the same average time. Mean differences between temperatures were determined using Fisher’s protected least significant differences.

## Results

Water quality parameters (total dissolved solids, ammonia, pH, dissolved oxygen, alkalinity, and conductivity) remained within ranges compatible with egg and larval rearing. Mean water hardness for all treatments was 301.43 mg/L as CaCO_3_ (214–344 mg/L as CaCO_3_). Total dissolved solids ranged between 0.0000–0.7203 mg/L. Mean total ammonia concentration ranged between 0.021–0.688 ppm as NH^3-^N. Mean pH was between 7.96–8.49 in all treatments. Conductivity ranged between 512–675 μS/cm, and dissolved oxygen was between 7.2–9.3 mg/L. Alkalinity ranged between 196–288 mg/L as CaCO_3_.

For the bighead carp 2011 study, there was a high degree of egg and larval abnormalities and mortality in the “cold” treatment, and no larvae from that treatment survived past the gas bladder emergence phase. There was no corresponding increase in abnormality or mortality for the “warm” treatment, where large numbers of larvae were successfully cultured past the gas bladder inflation stage. All water quality indicators, other than temperature and dissolved oxygen, were not significantly different between the two treatments. The holding temperature of the spawning fish (temperature = 25.0°C) was significantly higher than the “cold” treatment, and we suspect that the eggs experienced thermal shock and thus did not develop normally [21]. Mortality and abnormal development in all other instances were low, although not quantified.

### Developmental rates

CTU requirements and T_min_ were similar between species (Tables 1 and 2). For embryonic stages, a T_min_ of 12.9° C for bighead carp (r^2^ = 0.9968, root MSE = 5.325) and 12.1° C for silver carp (r^2^ = 0.9948, root MSE = 6.4246) were found to produce the lowest variance. T_min_ for larval stages increased to 13.3° C for silver carp (r^2^ = 0.9621, root MSE = 80.51) and 13.4° C for bighead carp (r^2^ = 0.9708, root MSE = 58.467).

**Table 1:** Cumulative thermal Units (CTUs) for each stage of embryonic development in bighead carp and silver carp for a T_min_ of 12.1° C for silver carp and 12.9° C for bighead carp.

	Silver carp		Bighead carp	
Stage	Mean	Standard deviation	Mean	Standard deviation
1) 1-cell	0.30	0.47	0.36	0.43
2) 2- cell	9.05	1.83	7.34	1.04
3) 4-cell	12.67	1.24	12.13	1.78
4) 8- cell	16.49	1.88	16.05	2.05
5) 16-cell	19.91	2.21	19.40	2.21
6) 32-cell	24.61	1.73	23.23	0.43
7) 64-cell	29.01	1.65	28.54	3.00
8) 128-cell	35.22	2.18	32.20	1.52
9) Morula	41.26	4.49	39.57	1.11
10) Early blastula	46.15	4.92	44.45	1.14
11) Mid-blastula	61.52	6.33	56.97	7.18
12) Late blastula	71.21	8.07	66.41	4.88
13) Early gastrula	83.50	6.05	75.30	0.64
14) Mid-gastrula	97.45	3.86	96.48	1.90
15) Late gastrula	106.14	3.81	108.87	4.20
16) Neurula	124.44	7.46	120.73	6.05
17) Blastopore closure	131.47	6.52	128.60	5.76
18) Somite appearance	139.94	2.40	136.51	5.17
19) Optic vesicle	150.23	2.97	146.71	6.25
20) Optic primordium	160.88	6.63	154.10	4.84
21) Olfactory placode	170.02	8.18	161.99	8.47
22) Tail bud	178.54	8.49	169.02	8.05
23) Tail vesicle	187.32	10.56	176.18	5.94
24) Otic capsule	191.44	7.25	186.39	7.21
25) Caudal fin	201.44	5.47	203.06	5.11
26) Lens formation	209.15	5.48	210.17	5.87
27) Muscular effect	224.50	5.28	223.45	9.91
28) Heart rudiment	237.96	11.77	237.41	6.71
29) Otolith formation	253.21	13.48	254.49	6.32
30) Heart pulsation	265.46	11.53	270.32	8.70

**Table 2:** Cumulative thermal Units (CTUs) for each stage of larval development in bighead carp and silver carp for a T_min_ of 13.3° C for silver carp and 13.4° C for bighead carp.

	Silver carp		Bighead carp	
Stage	Mean	Standard deviation	Mean	Standard deviation
31) Hatching	248.83	22.09	274.66	23.52
32) Pectoral fin	337.58	30.82	337.92	17.65
33) Gill arch	431.54	43.82	392.60	65.24
34) Xanthic eye	487.57	31.77	503.10	88.10
35) Gill filaments	562.30	17.16	633.16	49.72
36) Melanoid eye	697.73	33.44	764.90	52.80
37) Gas bladder emergence	891.53	97.04	963.75	79.72
38) Gas bladder inflation	1084.59	135.79	1161.07	36.72
39) Yolk-sac absorption	1318.65	187.16	—	—

### Egg size

Mean size of unfertilized eggs before water-hardening was 1.4 mm for silver carp and 1.5 mm for bighead carp, with almost no perivitelline space. Eggs began swelling, with concomitant increase in perivitelline space, immediately upon exposure to water. The initial period of swelling seems to be completed by four hours, regardless of temperature or species.

**Figure 1:**
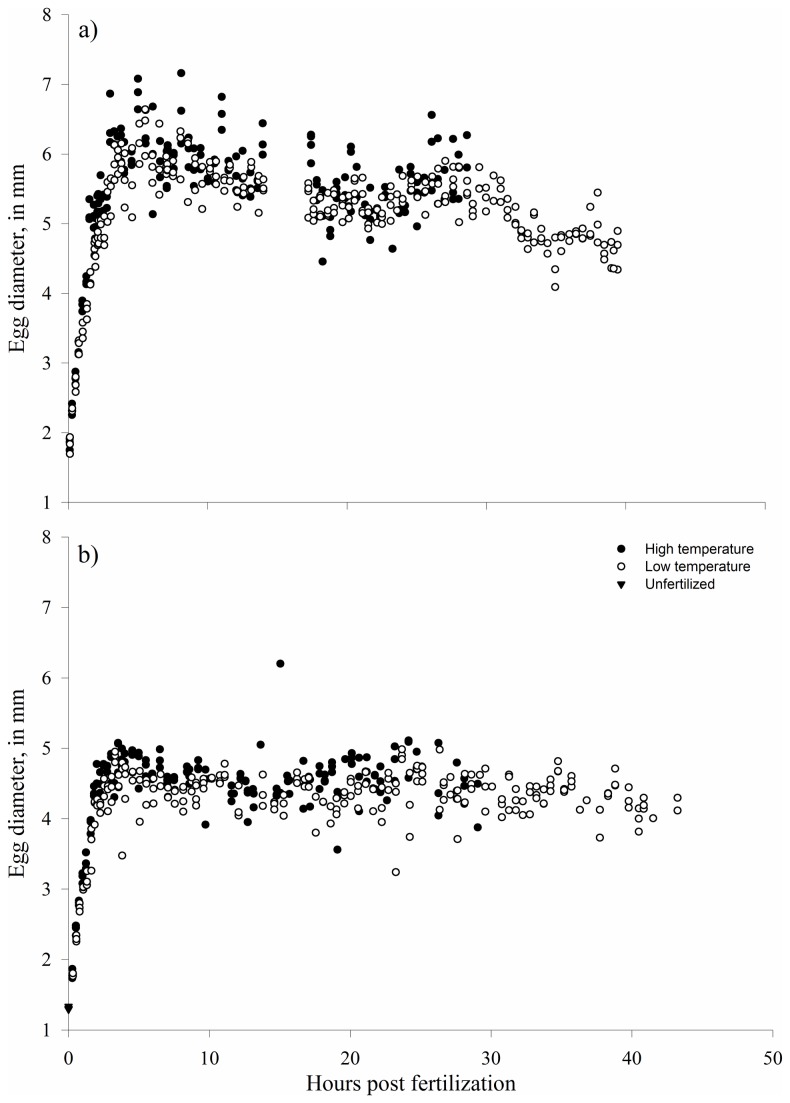
Egg diameters (mm) in different temperatures over time for a single experiment of bighead carp (a) and silver carp (b). The other experiments followed the same general trends.

In all bighead carp experiments and two of the silver carp experiments, eggs in the post-water hardening period were significantly larger in the “warm” treatment than the “cold” treatment. In 2011, mean size of bighead carp eggs in the “warm” treatment was 5.72 mm, whereas mean size was 5.35 mm in the “cold” treatment (p < 0.001). In the first 2011 silver carp experiment, mean size of eggs in the “warm” treatments was 4.59 mm, while mean size of silver carp eggs in the “cold” treatments was 4.38 mm (p < 0.001). In the second 2011 silver carp experiment, mean size of eggs was 4.71 mm in the “warm” treatment and 4.70 mm in the “cold” treatment (p > 0.05). In all trials, silver carp eggs ranged from 3.2–6.4 mm after water hardening (mean = 4.45 mm, SD = 0.36), and bighead carp ranged from 3.6–7.2 mm (mean = 5.4 mm, SD = 0.50) after water hardening (Figure 1).

### Behavior

Temperature within swimming tubes varied over diel periods from 18.5 to 26.0°C during the silver carp experimental periods. For the bighead carp experiment, temperature fluctuated between 22.5 and 28.0°C. There was no observed difference between top and bottom temperature of the swimming tubes. Upward vertical swimming, with alternating periods of sinking, began immediately after hatching. Larvae would usually swim upward for a few to several seconds. When upward swimming ceased, the larvae would sink, without swimming, in a head-downward position. The sinking period was generally a few seconds longer than the upward swimming period. This behavior continued until the end of the melanoid eye stage/beginning of the gas bladder emergence stage, at which time horizontal swimming began. During each developmental stage, individual larvae often stayed within a 50-cm range, with most ascents and descents covering only a portion of that range.

**Figure 2:**
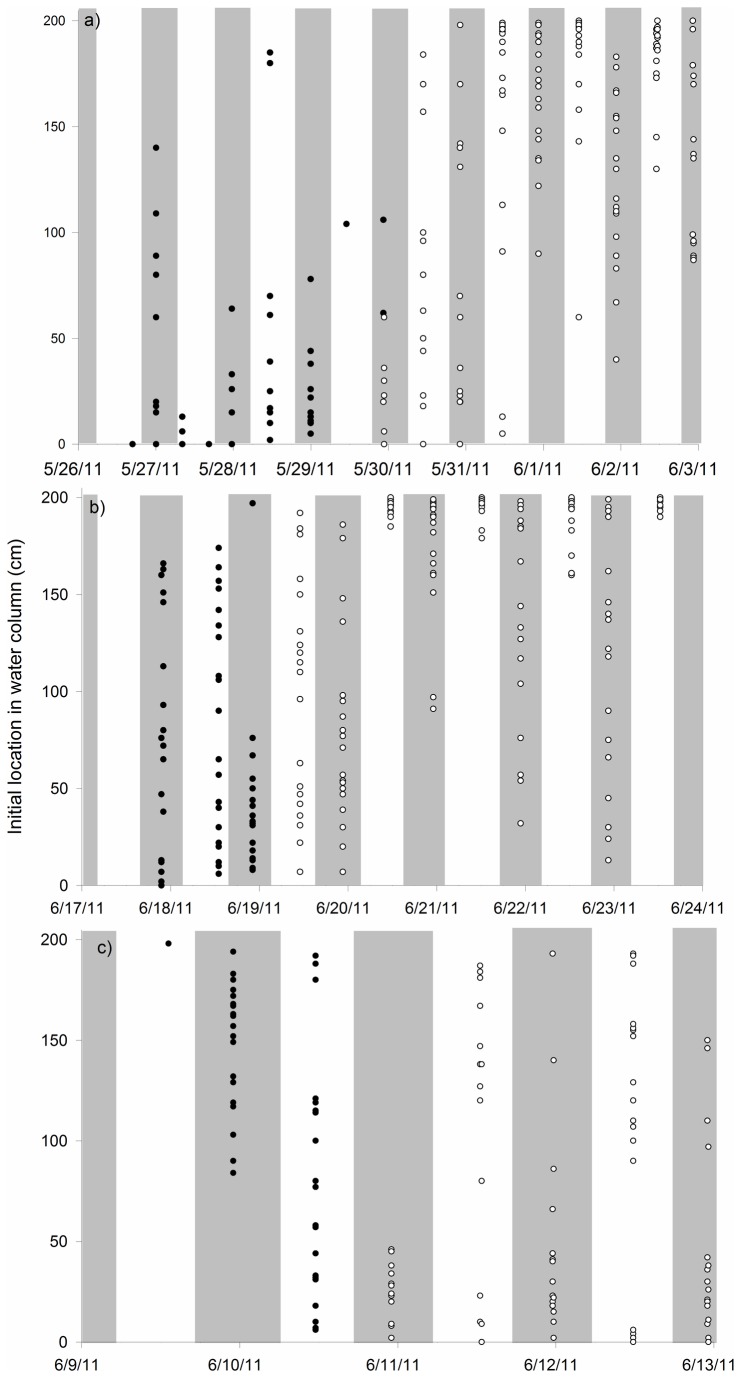
Initial water column location (cm from bottom) for silver carp larvae (a,b) and bighead carp larvae (c) over time. Dark circles represent vertical swimming and open circles represent horizontal swimming. Shaded areas represent night periods.

Water column position of the fish changed over the course of the study. Bighead carp started high in the water column, and gradually shifted to generally low positions during the night and higher positions during the day (Figure 2a), whereas silver carp started lower in the water column, and gradually shifted to the highest portion of the water column during the day. Following the onset of horizontal swimming, night positions were also high, but tended to occupy a larger range of water column positions (Figure 2b and 2c). Direct light from flashlights during dark period observations was avoided, but if light struck the larvae directly, it often caused larvae to sink, especially after the melanoid eye stage.

Initial measurements of bighead carp vertical swimming had a mean ascent rate of 3.75 cm/s (n=19, SD=0.798) at 39.5 hours post fertilization (HPF; hatching-rudimentary pectoral fin stage), before increasing to 5.69 cm/s (n=8, SD=1.12) at 51.9 HPF (gill arch-xanthic eye stage). Bighead carp had a mean descent rate of 2.065 cm/s (n=16, SD=0.422) at 39.5 HPF, before decreasing to 1.58 cm/s (n = 5, SD=0.23) at 51.9 HPF (gill arch-xanthic eye stage). Fish had begun horizontal swimming by the beginning of the gas bladder emergence stage, and shortly thereafter inflated their gas bladders. After gas bladder inflation, fish were capable of holding their position within the water column without active swimming. Carps are physostomes, and some physostomes must initially inflate the gas bladder by gulping air at the surface [22], but we did not observe this activity in bigheaded carps.

Silver carp in the first experiment had an initial mean ascent rate of 0.36 cm/s (n=2, SD=0.0405) at 52.4 HPF (hatching stage) and increased to 4.77 cm/s (n=11, SD=1.9) at 107.6 HPF (gill filament-melanoid eye stage). In the second silver carp experiment, initial mean ascent rate was 2.09 cm/s (n=10, SD=0.065) at 83.8 HPF (xanthic eye stage), which increased to 5.99 cm/s (n=9, SD=1.67) at 108.5 HPF (gill filament-melanoid eye stage). Silver carp in the first experiment had an initial mean descent rate of 0.83 cm/s (n=2, SD= 0.236) at 52.4 HPF and increased to 1.27 cm/s (n=8 SD=0.61) at 107.6 HPF (gill filament-melanoid eye stage). In the second silver carp experiment, initial mean descent rate was 2.39 cm/s (n=9, SD=0.963) at 83.8 HPF, which increased slightly to 2.54 cm/s (n=7, SD=2.09) at 108.5 HPF (gill filament-melanoid eye stage).

## Discussion

The results of this study provide the means to improve models that use minimum drift distance to assess potential of the establishment of bigheaded carps, such as [4]. The results can also be used in models to determine the location and timing of bigheaded carp spawning events, as in Deters et al. [15]. There are other potential uses of these data on egg and larvae development rate and larval behavior, such as prediction of the river reach at which larvae might be expected to leave the drift and occupy off-channel nursery habitat, larval growth studies, and aquaculture needs.

The observations of the time required for water hardening are also relevant to assessments of establishment potential, because smaller, more dense, eggs require higher turbulence to remain suspended in the drift. Eggs will require somewhat more turbulent water for approximately four hours after fertilization (regardless of temperature) to remain suspended, but most of that change in sinking rate, and thus the requirement for increased turbulence, will occur over the first hour.

### Developmental rates

Though there are a number of critiques of CTU methods [23,24], Hamel et al. [25,26] found the CTU method (referred to as degree days) to predict incubation times with sufficient accuracy for most fishery management needs. The CTU method of quantifying developmental rate requires that temperature be relatively constant (i.e., less than 2° C of variation), as large temperature fluctuations can affect survival, growth rate, developmental rate, and behavior [18,25,27,28]. The CTU method is also less accurate at temperatures near limits of thermal tolerances [24,27,29], although these rarely occur in natural settings [25]. Temperatures in this study were not near either the thermal maximum (39° C) or minimum (11-18°C) [13,30–32]. For successive stages, thermal requirements increase with morphological differentiation [14] and there is not a constant T_min_ that can be used for every stage of development. We found the degree of change small enough to use one T_min_ for all embryonic stages and another for larval stages. T_min_ is influenced by maternal history, acclimation temperature, stock and population differences and other environmental factors [25,26,29,32,33]. Values are best-fit data based on all temperature treatments for each species and are best used as an estimate for fish and conditions found in waterways of central North America.

Developmental rates are extremely temperature dependent, and small changes in temperature can have large effects on developmental time. A temperature difference of less than three degrees Celsius resulted in a 15-17 hour difference in hatching time. Larval fish reached the gas bladder inflation stage 2-3 days later in cooler temperatures than in warmer treatments. Temperature is critical for computing length of river required to keep eggs from sinking to the bottom until they hatch; a 3° C decrease in temperature (assuming constant river velocity) would increase that length by 50% or more. Similarly, a temperature difference of only 3 degrees would add 2-3 days to the time between fertilization and development to the larval stages which begin to leave the drift and move into low velocity off-channel habitat.

### Egg size

Water-hardened egg size was generally smaller here than reported by Yi et al. [7] and Murty et al. [6], though it was similar to values reported by Soin and Sukhanova [34]. This may be related to many factors, including maternal effects, ionic composition of water, water hardness, and environmental gradients [13,35–40]. Water in this study was hard (greater than 250 mg/L as CaCO_3_), therefore previously described effects of soft water [39,40] would not be observed.

Burt et al. [33], state that maternal/paternal identity influenced offspring responses to temperature in most studies. Although there was no direct test for parental influences in this study, the limited data seems to indicate an effect. Similar to Wu and Tan [38], egg size seemed to increase with maternal size, especially in silver carp. Eggs from larger fish (e.g. the second silver carp experiment in 2011) also showed a smaller or no effect of temperature on diameter.

Egg size is a useful character for general identification of Asian carps (bigheaded carps, as well as grass carp, *Ctenopharyngodon* *idella* , and black carp, *Mylopharyngodon* *piceus* ) in North America [15,41]. Size and morphology of these eggs are unique compared to native species, although differences are insufficient for species-level identification amongst the Asian carps. Egg size changed little after water hardening; therefore it is not useful as an aging tool in the post-water hardened period.

In a study of artificial eggs designed to mimic semi-buoyant cyprinid eggs, Dudley and Platania [42] note that particle size noticeably affects travel rates and settling times. The general trend of larger egg diameter with temperature shows that dispersal rates are affected by water temperature beyond affecting developmental rates. Temperature also affects the density and viscosity of water, so the swimming behavior and buoyancy of larvae and eggs can also be affected [18], which may affect dispersal. This is more likely to be a factor when considering a wider range of temperatures than examined in this study.

Water hardening took place over approximately four hours, with most of the change in size occurring in the first part of that period (Figure 1). This rapid change in size is important because by taking in water, the density of the eggs becomes closer to that of water. The size and density of the egg control the sinking rate and the propensity of the egg to remain suspended in the water column by turbulence [15]. Thus, by taking on large volumes of water and increasing dramatically in size, bigheaded carp eggs can be transported in rivers of lower velocity and turbulence. Before water-hardening, the eggs are smaller and more dense, which probably explains the preference of bigheaded carps for spawning in areas of high turbulence [43–45]. It should be possible to establish minimum required turbulence for transport of eggs in the pre- and post-water-hardening periods, which is critical in assessing potential of bigheaded carp establishment and determining spawning locations for potential control efforts.

### Swimming behavior

Chapman and George [13] previously noted the vertical swimming behavior that occurs prior to gas bladder development in bigheaded carps. However, this research is the first assessment of swimming speed, sinking rates, and water-column location selection by bigheaded carps.

Temperature fluctuations within swimming tubes were large enough that stage of larvae cannot be consistently predicted by the CTU model. Thus, all information on stage is based on observational data and is less precise than laboratory CTU data. Although other research [46–48] has shown an effect of temperature on larval behavior and swimming performance, this experiment was not designed to do so.

Nevertheless, there is a clear general trend of increasing swimming ability with ontogeny. Vertical swimming started immediately after hatching, although speed, frequency, and duration increased over time. Vertical swimming behavior immediately after hatch disputes the idea of passive drift for bigheaded carp larvae. Although all measurements were made in an environment lacking any flow, larval bigheaded carps seem to show enough swimming ability to interact with currents and influence dispersal potential from an early developmental stage.

It is believed that early life stages of Asian carp require a sufficient velocity or turbulence, or the embryos and larvae will sink to the bottom and die [3]. The early swimming behavior displayed by bigheaded carps suggests that current is not required for survival of larvae, though it may be necessary for embryonic stages. Estimations of minimum river length should thus probably be computed using time between fertilization and hatching.

Because initial swimming is entirely vertical, larvae in areas with low or absent current may not be able to advance to typical post-larval habitats until they develop more fully. In collections of larvae on the Missouri River and its tributaries, only larvae older than gas bladder emergence (associated here with the beginning of horizontal swimming) were collected in low-velocity off-channel habitats [15]. Combined with models of river hydraulics and known spawning locations of bigheaded carps, CTU values from this study could be used to determine existence of suitable habitats in the vicinity of the first lateral movements. This can be used in assessing the risk of establishment or determining sampling locations for juvenile bigheaded carps.

In the Great Lakes, flooded tributary mouths often provide freshwater estuary conditions with abundant wetland habitats [49] that might be used as nursery habitats by juvenile bigheaded carps. If larvae are washed into these areas prior to the beginning of lateral swimming, they would likely be retained within the estuary until lateral swimming begins, providing them with access to high quality nurseries. However, rates of predation on the vertically swimming larvae in the flooded tributary mouths and other factors influencing survival under these unique conditions have not been researched.

Early detection of bigheaded carp spawning or recruitment is vital for rapid response efforts against these invasive species. This early detection of bigheaded carp spawning will be most practical by capture of eggs and larvae, because later stages are net-shy and locate within shallow vegetated habitats that are difficult to access and sample. The water column distribution of these larvae will be useful for monitoring efforts to detect successful spawning events.

One might speculate that swimming to maintain position in the water column would be an excessive energy demand on larvae, compared to being kept from sinking to the bottom by turbulence, and thus important in the calculation of minimum river velocity and turbulence for recruitment. We do not believe that this is an important factor. Bigheaded carps do not begin feeding until their mouth parts fully develop, after inflation of the gas bladder and beginning of the ability to maintain vertical position without swimming. Survival was high in all experiments and treatments except in one case of temperature-shocked eggs. These fish, which began vertical swimming and water column depth selection in calm water immediately after hatching, did not starve and began to feed normally when food was offered after termination of this study.

When larval fish began horizontal swimming, water column position selection became more pronounced (Figure 2). Differing diel vertical distribution of larvae beginning with development of the melanoid eye suggests a phototactic response. Phototaxis is common in early development among many species of fish [50,51], and could be advantageous as a means of predator avoidance in turbid rivers where bigheaded carps tend to flourish. In fluvial systems, fastest current velocity tends to occur near the water surface [52], so a positive phototactic response where larvae were distributed near surface water would have the effect of increasing current exposure and maximizing dispersal potential [53]. It should be noted that many or most rivers where bigheaded carps spawn are turbid, and these exposure chambers were not. It is unknown if the observed diel movements would occur in turbid rivers. It is also unclear whether the sinking of the larvae from direct light is a startle response or some form of phototaxis. While this was not directly addressed in this study, a phototactic response suggests that light traps may be suitable gear for capturing later larval stages of bigheaded carp.

Deters et al. [15] used river drift velocity and egg capture location was combined with the CTU model developed here to assess locations of bigheaded carp spawning in the Missouri River. The CTU model can also be used to improve drift models to assess the possibility that rivers could be used by bigheaded carps for reproduction, such as in tributaries of the Great Lakes or reservoirs [4,54]. Murphy and Jackson [55] used this CTU model in drift models to assess the potential of bigheaded carp establishment in four tributaries of Lake Erie and Lake Michigan (the Sandusky, Maumee, St. Joseph and Milwaukee Rivers). They found that under some conditions in modeled rivers, distances as short as 25 km could be adequate for transport of eggs until hatching, and that all four rivers had sections with adequate characteristics for successful transport of bigheaded carp eggs. Inclusion of behavioral data, suggesting that the fish are capable of selecting water-column location under low velocity conditions, can help to further refine drift models, and provide information to plan the timing and location of larval sampling and to interpret larval sampling data. Information on swimming velocity would also be useful in determining what level of turbulence is required to overcome the swimming ability of larvae, and therefore important in modeling efforts to understand larval drift and planning sampling of larval fish.
